# Pulmonary Spindle Cell Carcinoma: As Rare as a Hen’s Teeth

**DOI:** 10.7759/cureus.54266

**Published:** 2024-02-15

**Authors:** Rudra P Samanta, Srikant Agarwal, Ruchi Agrawal, Kaushik Saha, Susmita Sengupta

**Affiliations:** 1 Department of Pulmonology, Tata Main Hospital, Jamshedpur, IND; 2 Department of Pathology, Manipal-Tata Medical College, Jamshedpur, IND; 3 Department of Pathology, Tata Main Hospital, Jamshedpur, IND

**Keywords:** biopsy, pneumonia, pulmonary, spindle cell carcinoma, lung mass

## Abstract

This case is about a 70-year-old man who presented with symptoms and laboratory reports that indicated differentials toward an infectious disease (pneumonia and tuberculosis). A lung mass was found in his chest X-ray and in the computerized tomography (CT) scan of his thorax. A biopsy was taken from the lung mass, and histopathological examination and immunohistochemical staining of the biopsy were done. The results revealed the presence of spindle cell carcinoma (SpCC) with vimentin and cytokeratin positivity. Spindle cell lung cancer is a rare type of non-small cell lung carcinoma, for which all available research indicates a poor prognosis. Due to the rarity of diagnosis, there is a dearth of information about the epidemiology and overall survival of affected patients.

## Introduction

Pulmonary sarcomatoid carcinomas are rare, high-grade, poorly differentiated carcinomas and consist of a small subset of non-small cell lung carcinoma. They form 0.2-0.3% of all kinds of lung cancers [1]. Based on histological features, the WHO classification categorizes them into pleomorphic carcinoma, spindle carcinoma, giant cell carcinoma, carcinosarcoma, and pulmonary blastoma. Spindle cell carcinoma (SpCC) is a rare histological variant of sarcomatoid carcinoma. It consists of a pure population of spindle-shaped tumor cells. According to available literature, pulmonary SpCC involves mostly the periphery of the lungs [2]. Very less is known about the clinical presentation, disease progression, and management of SpCC. Survival outcomes in spindle cell cancer are contingent upon variables, such as the patient’s age, malignancy, and the stage of the disease at the time of diagnosis [3].

This patient had a mass, which was circular in shape, of approximately 10 cm × 9.6 cm, situated in the upper and middle zone of the right lung. Histopathological findings from a biopsy taken from this mass indicated the presence of spindle-shaped cells arranged in bundles, along with a few multinucleated tumor giant cells, as well as atypical mitosis along with ongoing apoptosis. Eventually, immunohistochemical staining revealed the diagnosis to be SpCC with a combined vimentin and cytokeratin positivity. Since this was a rare presentation, we hope that this case can contribute to the existing literature and ultimately pave the path toward research aiming at increasing life expectancy and obtaining a better prognosis in patients with pulmonary SpCC.

## Case presentation

**Figure 1:**
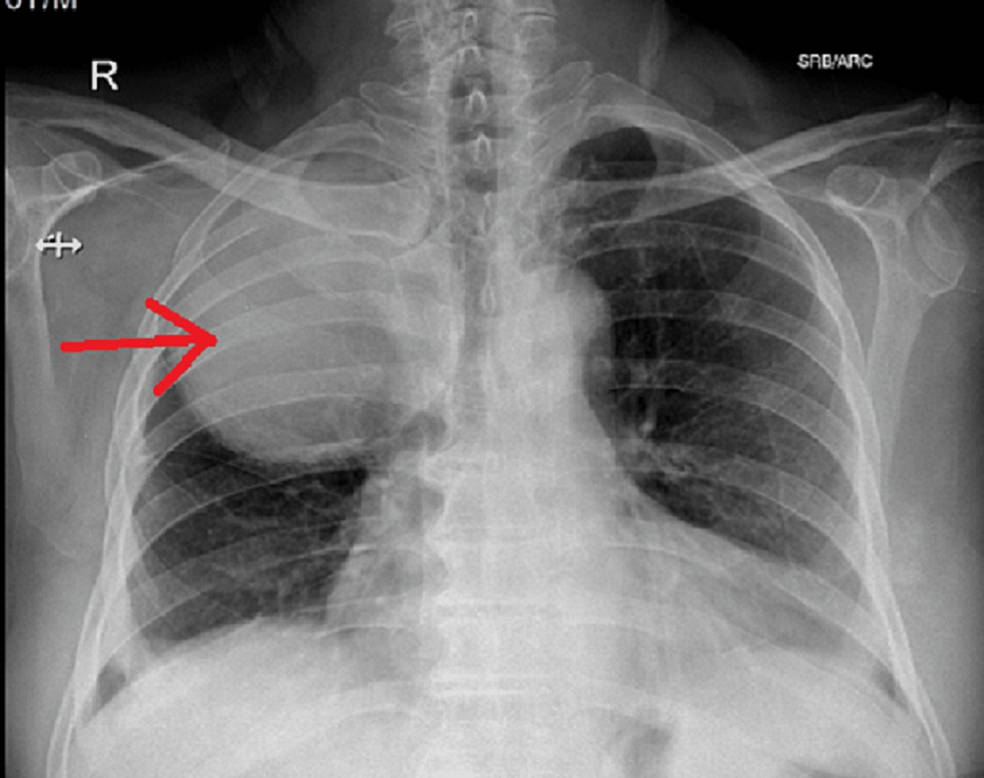
Chest X-ray (posteroanterior view) It shows a circular opacity occupying the upper and middle zone of his right lung (red arrow).

A 70-year-old male was admitted with complaints of continuous recurrent high-grade fever for a month, accompanied by cough and hemoptysis on and off. He also had right-sided chest pain for 10 days prior to admission. He was alert and oriented at the time of examination. The patient had a temperature of 102 degrees Fahrenheit on admission, while the blood pressure, oxygen saturation, and pulse rate were within normal ranges. He had no complaints of dyspnea. The patient reported no history of smoking nor of any alcohol intake. The patient had no history of exposure to second-hand smoke nor any workplace exposure to smoke or dust. He had a history of hypertension and was on losartan for 10 years. On examination of his respiratory system, he was tachypneic, with increased use of accessory muscles of respiration and increased vocal fremitus in his right infra-scapular area and right inter-scapular area, and auscultation revealed reduced breath sounds in the right infra-scapular and inter-scapular areas and crackles in the bilateral infra-scapular areas. His chest X-ray showed a circular opacity occupying the upper and middle zone of his right lung (Figure 1). 

**Figure 2:**
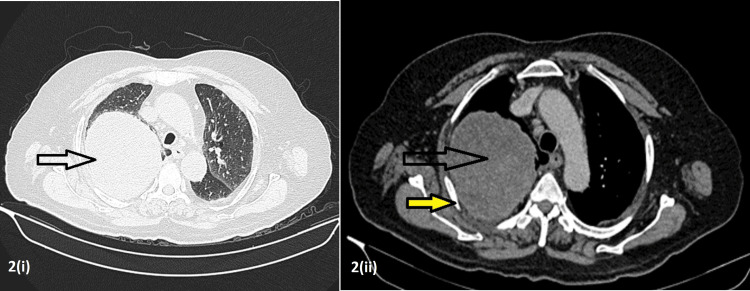
Contrast-enhanced computed tomography (CECT) of the thorax It shows a well-defined heterogeneously enhancing mass in the right upper lobe with areas of necrosis approximately 10.7 x 9.1 x 9 cm (black arrows in both images 2(i) and 2(ii)). Atelectatic changes were also seen in the adjacent lung parenchyma, which were abutting and compressing the right upper lobe and segmental bronchi. Extension of the mass into the chest wall was seen in the posterior aspect with erosions of the third rib (yellow arrow in image 2(ii)).

A contrast-enhanced computed tomography (CECT) of the thorax showed a well-defined heterogeneously enhancing mass in the right upper lobe showing areas of necrosis approximately 10.7 x 9.1 x 9 cm. Atelectatic changes were also seen in the adjacent lung parenchyma, which were abutting and compressing the right upper lobe and segmental bronchi. Extension of the mass into the chest wall was seen in the posterior aspect with erosions of the third rib (Figure 2).

A CT-guided biopsy from the mass was done, and the sample was sent for histopathological examination. The histopathological section showed a linear core of the tissue having a proliferation of spindle cells arranged variably in fascicles, along with a haphazard pattern and focal swirling. Cellular density was homogenous throughout the lesion and punctuated by areas of coagulative necrosis. Focal myxoid change was also seen. Individual spindle cells showed five granular chromatins. A few multinucleated tumor giant cells, atypical mitosis, and apoptosis were also seen.

**Figure 3:**
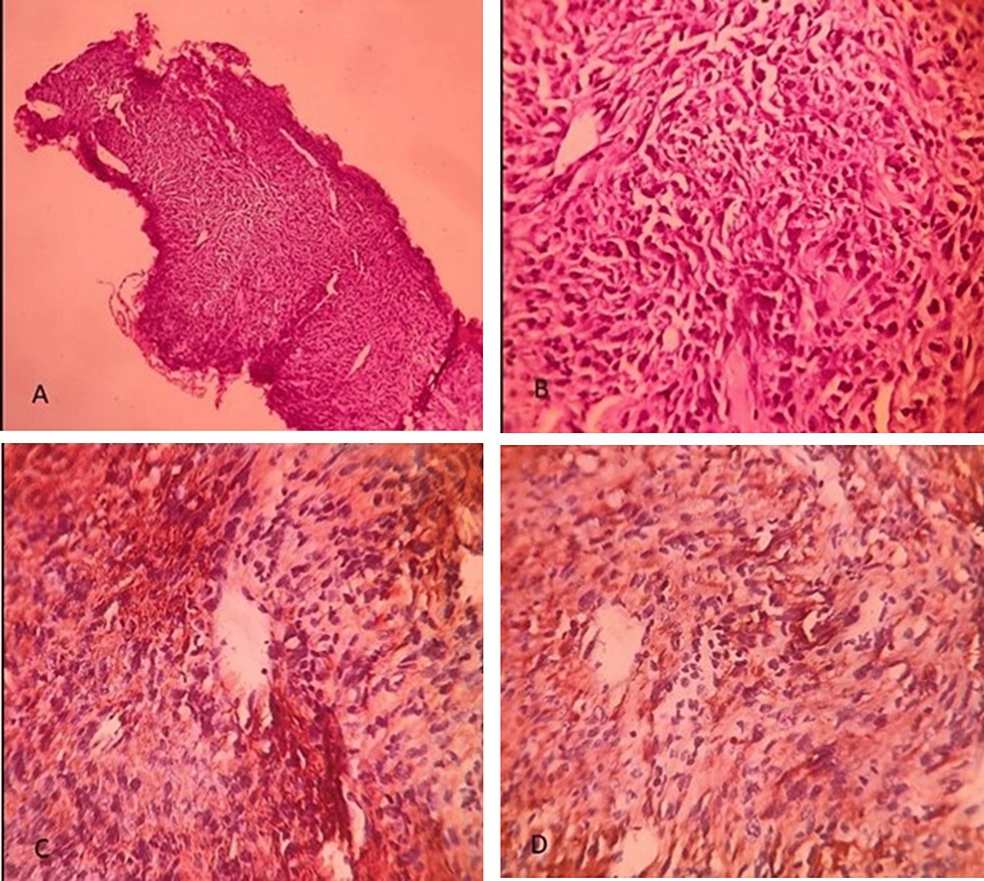
Immunohistochemical staining of the biopsy of the lung mass A: Lung core showing diffuse proliferation of spindle cells with a haphazard pattern; no epithelial element was noted (hematoxylin and eosin (H&E), 100x). B: Higher magnification shows spindle cells with moderate pleomorphism, open chromatin, variably prominent nucleoli, and scant cytoplasm (H&E, 400x). C: Cytokeratin stain shows diffuse positivity in the tumor cells (AE1/AE3, DAB, 400x). D: Vimentin stain shows diffuse uptake in the neoplastic cells (vimentin, diaminobenzidine (DAB), 400x).

For further analysis, immunohistochemical staining of the lung tissue sample was done. It showed high positivity for the immunohistochemical markers pan-cytokeratin (CK) and vimentin. Retained expression was detected for the marker integrase interactor-1 (INI-1). Markers, such as smooth muscle actin (SMA), CD34, CD117, desmin, p63, P40, thyroid transcription factor TTF-1, NAPSIN-A, and SRY-box transcription transcription factor, i.e., SOX-1, were negative. These findings confirm the diagnosis of SpCC (Figure 3).

Treatment plans for this carcinoma are limited. They include surgical resection, chemotherapy, and radiotherapy. The patient had undergone four cycles of platinum-based chemotherapy and had refused to opt for surgery. Unfortunately, the patient passed away after the fourth chemotherapy session.

## Discussion

SpCC of the lung is very commonly present peripherally. The presence of mitotic malignant spindle cells and hyperchromic nuclei with absent pleomorphic or giant cells on biopsy confirms the diagnosis. In SpCC, the immunohistochemistry shows positivity for cytokeratin and vimentin and the absence of desmin, S100 protein, α-smooth muscle actin, and CD34 cells. Programmed cell death ligand, i.e., PD-L1, is positive in nearly three-fourths of lung sarcomatoid cancers (pleomorphic, spindle cell, and giant cell). Even though PD-L1 expression directs toward a poor prognosis, it also makes the patient a candidate for targeted immunotherapy. The prognosis of SpCC is very poor. Only three out of 11 patients had a survival of more than two years in the case series of 11 patients by Qi et al. [4].

Management of eligible patients with localized disease consists of surgical resection with neoadjuvant/adjuvant chemotherapy. Based upon the Phase I/II KEYNOTE-021 trial results in non-small cell lung carcinoma patients, chemotherapy is administered to the patient [5]. This consists of a combination of pemetrexed and carboplatin, in addition to pembrolizumab. Our patient refused surgical resection and was started on chemotherapy.

Physicians can differentiate pulmonary SpCC from various spindle cell tumors by knowing the following details:

Inflammatory myofibroblastic tumor: Having a varied etiology, inflammatory myofibroblastic tumor is an uncommon lesion. First observed in the lungs, it mimics malignant neoplasms, such as metastatic sarcoma and sarcomatoid mesothelioma. It is also seen in various other areas, like the head and abdomen. It occurs more commonly in younger age groups, especially in children. It is a painless mass or swelling, which may be indurated and has symptomatology according to the site of origin. It shows positivity for smooth muscle actin (SMA), anaplastic lymphoma kinase (ALK), and desmin. It is treated by excision, steroids, radiotherapy, chemotherapy, or any combination of these treatments. A recently discovered treatment is the use of a CO_2_ laser for excision [6].

Malignant solitary fibrous tumor: This slow-growing tumor is most commonly known to be located intra-thoracically and is mesenchymal in nature. It is most commonly found in the anterior mediastinum among all other thoracic locations. It occurs in adults mostly in their fifth and sixth decades. It is mostly asymptomatic, but in some cases, it presents with non-specific chest pain, cough, or breathlessness. The size of this neoplasm varies from 7 to 10 cm, and malignancy is suspected usually when the tumor size exceeds 10 cm. More features indicating malignancy include recurrence, tumor necrosis or hemorrhage, and hypercellularity. Immunohistochemical findings show STAT6 positivity and epithelial membrane antigen (EMA) negativity. The mainstay of treatment is surgery, which may be combined with chemotherapy or radiotherapy [7].

Synovial sarcoma: It is a highly aggressive mesenchymal neoplasm with a high metastatic component. It has a characteristic t(X;18)(p11; q11) translocation that produces an SYT-SSX fusion gene. It most commonly occurs in adolescents and adults under 30 years of age. Usually, it presents as a deep-seated, hard, growing mass arising from the musculoskeletal systems in the extremities. Immunohistochemical features include expression of EMA and broad-spectrum CK, CD99, and TLE1. It is treated by surgical resection. Neoadjuvant chemotherapy is used in the pediatric population, but there is no agreed-upon consensus regarding the use of chemotherapy and radiation in adults [8].

Sarcomatoid mesothelioma: One of the subtypes of malignant pleural mesothelioma, it is a highly aggressive and rare form of mesothelioma and is associated with exposure to asbestos. It presents usually after the fifth decade of life. The pleura is the most commonly affected, with findings of large nodules on the pleurae. A serum biomarker used for malignant pleural mesotheliomas is the megakaryocyte potentiating factor [9]. Immunohistochemistry shows D2-40 and WT1 positivity.

Metastatic sarcoma: Metastatic sarcoma arises subsequent to the development of the primary tumor. The lungs are the most common area for metastases from soft tissue sarcomas [10].

## Conclusions

Pulmonary SpCC is a one of the rarer types of pulmonary malignancies. It is considered to have a very poor prognosis even after a combination of treatment protocols including surgery along with chemotherapy and radiation. There is reported to be only a 10% survival rate after two years of diagnosis. Hence, for a malignancy as rare as a pulmonary SpCC, increased efforts and diversified research are the need of the hour to improve prognosis and increase treatment efficiency. As is with any rare disease or rarely studied disease, it is of considerable importance that literature regarding it be available for study and discourse.
